# Evaluating the Biocompatibility of an Injectable Wound Matrix in a Murine Model

**DOI:** 10.3390/gels8010049

**Published:** 2022-01-09

**Authors:** Hatem Alnojeidi, Ruhangiz Taghi Kilani, Aziz Ghahary

**Affiliations:** 1 British Columbia Professional Firefighters’ Burn and Wound Healing Research Group, International Collaboration on Repair Discoveries (ICORD), Vancouver, BC V5Z 1M9, Canada; hatem.nojeidi@gmail.com (H.A.); ruhikilani@yahoo.com (R.T.K.); 2 Division of Plastic Surgery, Department of Surgery, University of British Columbia, Vancouver, BC V6T 1Z4, Canada

**Keywords:** wound healing, wound matrix, dermal scaffold, tissue engineering, injectable

## Abstract

(1) Background: Developing a high-quality, injectable biomaterial that is labor-saving, cost-efficient, and patient-ready is highly desirable. Our research group has previously developed a collagen-based injectable scaffold for the treatment of a variety of wounds including wounds with deep and irregular beds. Here, we investigated the biocompatibility of our liquid scaffold in mice and compared the results to a commercially available injectable granular collagen-based product. (2) Methods: Scaffolds were applied in sub-dermal pockets on the dorsum of mice. To examine the interaction between the scaffolds and the host tissue, samples were harvested after 1 and 2 weeks and stained for collagen content using Masson’s Trichrome staining. Immunofluorescence staining and quantification were performed to assess the type and number of cells infiltrating each scaffold. (3) Results: Histological evaluation after 1 and 2 weeks demonstrated early and efficient integration of our liquid scaffold with no evident adverse foreign body reaction. This rapid incorporation was accompanied by significant cellular infiltration of stromal and immune cells into the scaffold when compared to the commercial product (*p* < 0.01) and the control group (*p* < 0.05). Contrarily, the commercial scaffold induced a foreign body reaction as it was surrounded by a capsule-like, dense cellular layer during the 2-week period, resulting in delayed integration and hampered cellular infiltration. (4) Conclusion: Results obtained from this study demonstrate the potential use of our liquid scaffold as an advanced injectable wound matrix for the management of skin wounds with complex geometries.

## 1. Introduction

Chronic wounds are one of the most prevalent health conditions worldwide, causing disability and impaired quality of life for those affected, and producing a tremendous global economic burden [1,2]. Despite their varying etiologies, chronic wounds share common factors that contribute to their deficient healing potential, such as decreased cellular migration and proliferation, impaired vascularization, and reduced collagen production and extracellular matrix (ECM) synthesis [3,4,5]. These elements eventually lead to open, scaffold-less defects with an increased risk of infection and a raised likelihood of malignant transformation [6,7].

Injectable ECM-based biomaterials are now recognized as an advantageous therapy in the management of chronic wounds, and their application is preferred in the treatment of complex wounds with deep and irregular wound beds that are challenging to treat with conventional sheet-like scaffolds [8,9,10,11]. However, clinically available injectable scaffolds come with limitations that hinder their potential to effectively treat such wounds. They are mainly composed of a collagen-GAG component without any additives or nutrients to support scaffold hydration and stabilization once applied to the wound, leading to delayed and deficient integration with the host tissue. Additionally, their flowability is questionable, as they lack true fluidity once hydrated with saline (instead, forming a semiliquid or paste-like matrix) preventing the material from dispersing uniformly throughout the treated wound, resulting in unfilled void areas which contribute to delayed and insufficient repair [12,13].

**Figure 1:**
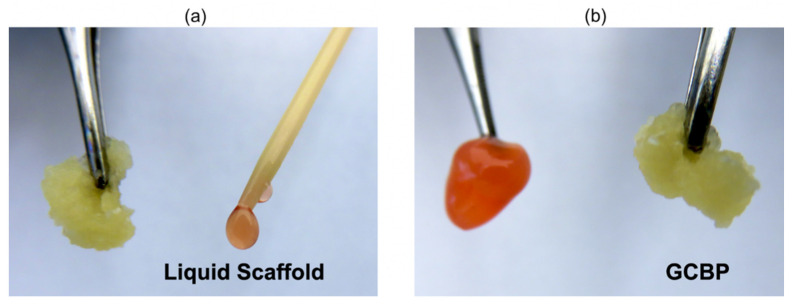
Liquid Scaffold and GCBP: (**a**) Immediately after being prepared from their powdered forms. (**b**) When kept at room temperature for 10–15 min. Liquid Scaffold forms a liquid matrix after reconstitution and transforms into a gel-like state when exposed to physiological temperatures, while GCBP forms into a slurry, semiliquid state that is maintained regardless of temperature changes.

Our research group has recognized these challenges and previously developed a novel, ECM-based injectable wound matrix designed for in-situ solidification for rapid tissue integration [14,15,16]. This injectable scaffold is composed of cross-linked bovine type I collagen and chondroitin sulfate, supplemented with polyvinyl alcohol (PVA), and contains optimum concertation of necessary amino acids, vitamins, and minerals required for cell growth and proliferations (Figure 1). We have previously examined the physical characteristics of our scaffold, including its tensile strength, fibril formation, thermal stability and collagenase digestion, and demonstrated maintained cell viability and morphology in vitro [14,15,16]. We have also showed that the topical application of our injectable scaffold in open wounds in a mouse model improved the healing outcome [16,17]. Moreover, we have considered the fact that epithelial cells interact with fibroblasts and modulate ECM synthesis and degradation [18]. Here, we have used our liquid scaffold in murine closed wounds where keratinocytes barely play any role in ECM modulation. The objective of this study was to evaluate the integration of the liquid scaffold following sub-dermal injection, and to compare the results to an injectable granular collagen-based product (GCBP), a product that is commercially available and clinically indicated for the treatment of tunneling wounds (Figure 1). To accomplish this objective, we developed a microsurgical technique to apply the scaffolds in direct contact with the mouse dorsal dermis, by dissecting between the dermis and the underlying, strongly attached panniculus carnosus muscle layer. We then histologically evaluated the interaction between the scaffolds and the host, by assessing the host response and quantifying the cellular infiltration into each scaffold.

## 2. Results and Discussion

### 2.1. In Vivo Integration of Scaffolds

**Figure 2:**
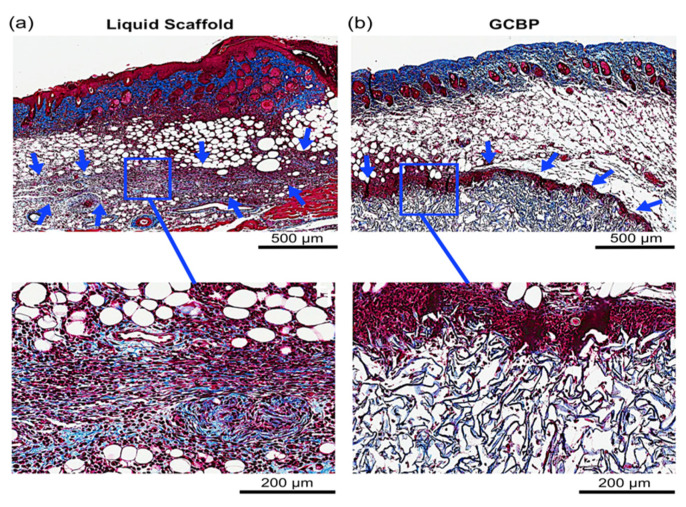
Histological evaluation after 1 week with MT staining. (**a**) Arrows point to the injected Liquid Scaffold. Higher magnification of Liquid Scaffold displays parallel and organized distribution of collagen fibers along the dermis, associated with a remarkable number of cells seen within the matrix. (**b**) Arrows point to the cellular layer surrounding GCBP. Higher magnification of GCBP shows a dense cellular layer encapsulating thick and randomly organized collagen fibers, with limited number of cells detected within the matrix.

**Figure 3:**
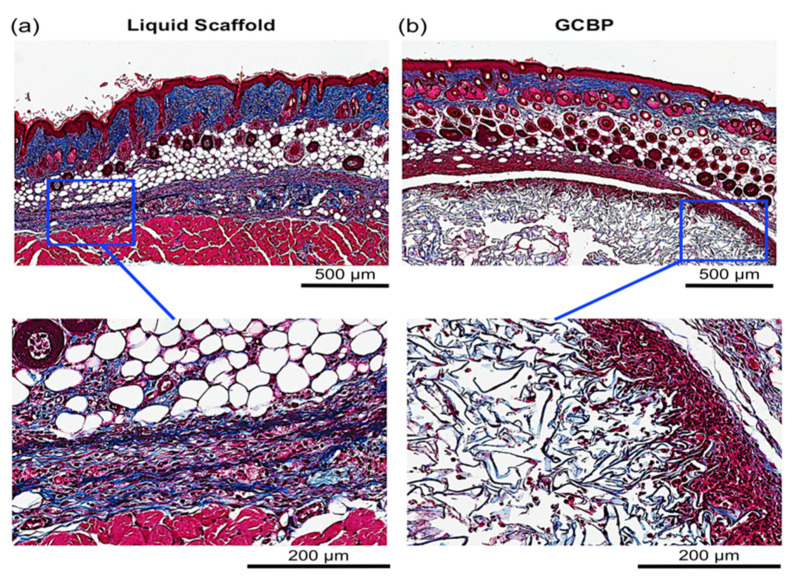
Histological evaluation after 2 weeks with MT staining. (**a**) Higher magnification of Liquid Scaffold shows denser blue staining compared to week 1. (**b**) Higher magnification of GCBP displays a persisting layer of highly cellular capsule surrounding the matrix, with continued limited cellular infiltration.

Each injectable scaffold exhibited a unique histological microstructure. Collagen fibers in Liquid Scaffold were distributed in a horizontal and organized fashion parallel to the dermis (Figure 2a and Figure 3a) while GCBP had thicker, randomly organized collagen fibers with large inter-fiber distances (Figure 2b and Figure 3b). After 1 week, Masson’s Trichrome (MT) staining showed early and efficient integration of Liquid Scaffold, apparent by (1) the lack of an obvious adverse foreign body reaction (FBR), and (2) the occurrence of an intense cellular infiltration into the scaffold, mostly of spindle-shaped, fibroblast-like cells (Figure 2a). In contrast, GCBP was associated with the formation of a discrete, dense and highly cellular layer surrounding the matrix, hampering cellular migration into the scaffold (Figure 2b). Results after 2 weeks revealed preserved cellular presence with a denser collagen staining in Liquid Scaffold (Figure 3a), while GCBP was persistently surrounded by the thick capsule-like layer, with continued limited cellular infiltration (Figure 3b).

### 2.2. Immunofluorescence Staining and Quantification

**Figure 4:**
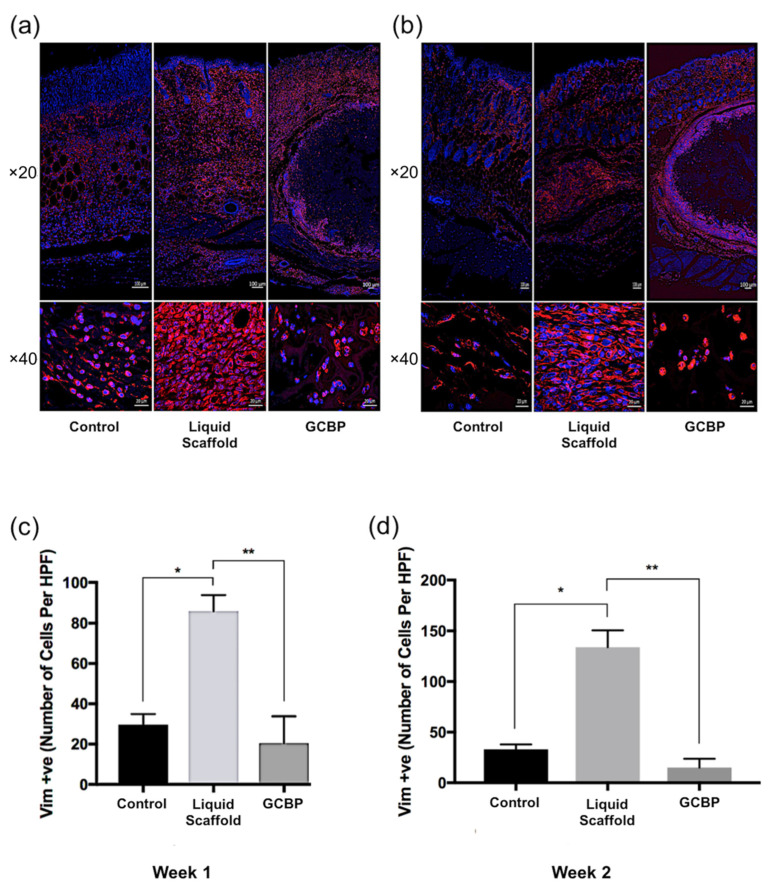
(**a**) Immunofluorescence staining of Vimentin-positive cells (red), counterstained with nuclear stain DAPI (blue) after 1 week. (**b**) IF staining of Vim+ cells after 2 weeks. (**c**) Quantification of Vim+ cells after 1 week. (**d**) Quantification of Vim+ cells after 2 weeks. Vim+ cells were the most dominant cell type in all groups at all time points. Liquid Scaffold had significantly higher numbers compared to GCBP and the control group (*, *p* < 0.05; **, *p* < 0.01). Scale bars in top and bottom rows = 100 µm and 20 µm, respectively.

**Figure 5:**
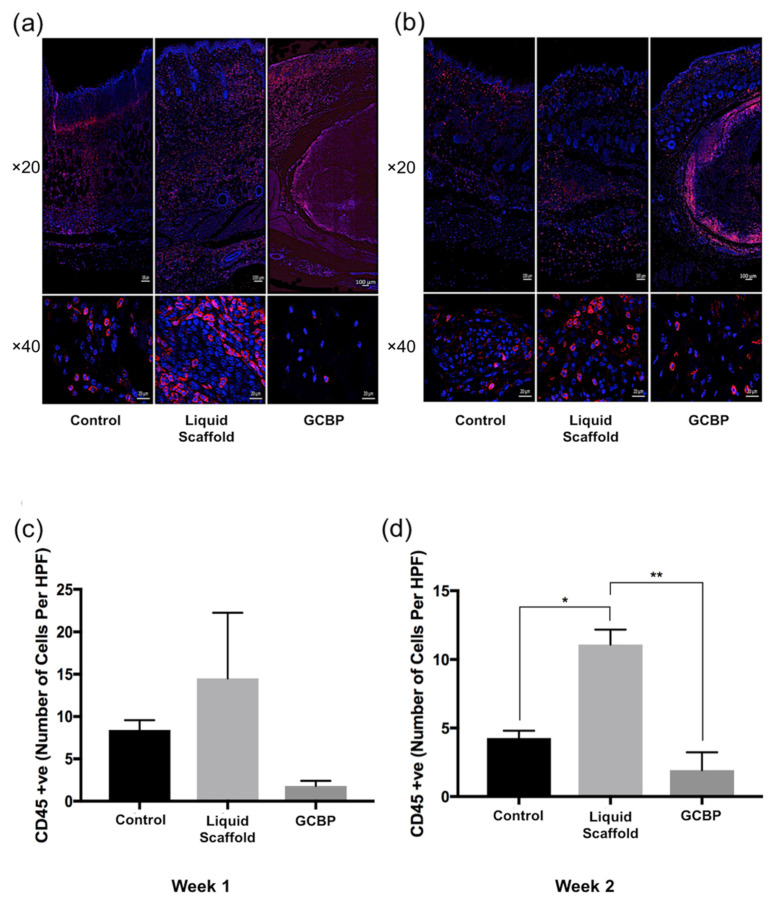
(**a**) Immunofluorescence staining of CD45-positive cells (red), counterstained with nuclear stain DAPI (blue) after 1 week. (**b**) IF staining of CD45+ cells after 2 weeks. (**c**) Quantification of CD45+ cells after 1 week. (**d**) Quantification of CD45+ cells after 2 weeks. CD45+ cells were the second most dominant cell type in all groups. Liquid Scaffold showed higher number of cells after 1 week, and significantly higher number after 2 weeks, compared to GCBP and the control (*, *p* < 0.05; **, *p* < 0.01). Scale bars in top and bottom rows = 100 µm and 20 µm, respectively.

**Figure 6:**
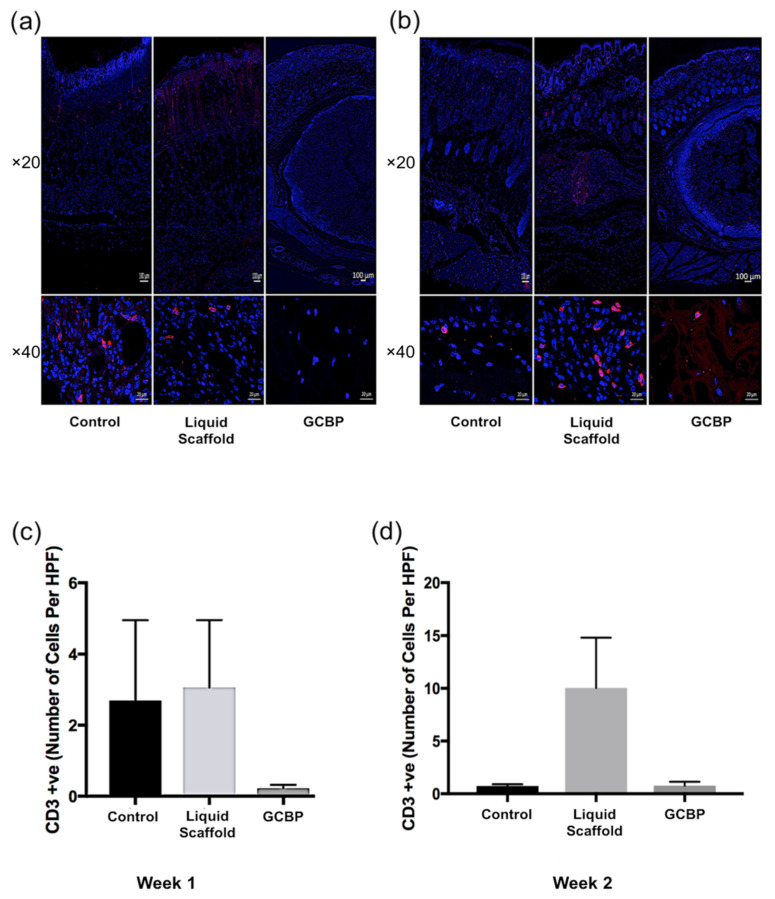
(**a**) Immunofluorescence staining of CD3-positive cells (red), counterstained with nuclear stain DAPI (blue) after 1 week. (**b**) IF of CD3+ cells after 2 weeks. (**c**) Quantification of CD3+ cells after 1 week. (**d**) Quantification of CD3+ cells after 2 weeks. The number of CD3+ cells was limited and with no significant differences among all groups. Scale bars in top and bottom rows = 100 µm and 20 µm, respectively.

Immunofluorescence staining (IF) was conducted after 1 and 2 weeks to examine cell phenotype and quantify in within scaffolds. After 1 week, cells infiltrating Liquid Scaffold were predominantly Vimentin-positive (Vim+) cells displaying the typical spindle-like fibroblast morphology, followed by lower number of CD45-positive (CD45+) and CD3-positive (CD3+) cells (Figure 4a, Figure 5a and Figure 6a, respectively). Cells seen within GCBP were mostly round-shaped Vim+ cells (Figure 3a), while cells within the capsule-like layer were predominantly spindle-like Vim+ cells and CD45+ cells (Figure 3a and Figure 4a). After 2 weeks, samples exhibited similar distribution of Vim+, CD45+, and CD3+ cells (Figure 4b, Figure 5b and Figure 6b, respectively). Quantification analysis revealed significantly higher number of Vim+ cells in Liquid Scaffold after 1 and 2 weeks compared to GCBP and the control group (*p* < 0.05) (Figure 4c,d). Moreover, in week 2 Vim+ cell number in Liquid Scaffold increased compared to week 1, while staying relatively the same in the other two groups (Figure 4c,d). CD45+ cells were higher in Liquid Scaffold after 1 week compared to GCBP and the control group, and significantly higher after 2 weeks (*p* < 0.05) (Figure 5c,d, respectively). Lastly, CD3+ cells were limited in number in all groups at all time points, with no significant differences among groups (Figure 6c,d).

### 2.3. Discussion

The ECM is a fundamental component of the cutaneous tissue and is indispensable for sustaining normal skin function and integrity, and for guiding wound healing following injury [19,20,21]. Over the past two decades, recognizing the vital roles of ECM components in orchestrating wound repair have led to a rising appreciation of the advantages of engineered ECM substitutes over conventional therapeutic approaches in the management of difficult-to-heal wounds [2,5,9,10,11]. Numerous ECM-mimicking products have been developed with varying compositions, differing structural characteristics, as well as unique manufacturing techniques. Of those, injectable scaffolds are the most suitable to apply on wounds with deep, irregular, and tunneled wound beds [22,23]. Unfortunately, clinically available, injectable products are scarce and lack true flowability [11,13,24]. To address these challenges, our research group has previously developed an injectable, collagen-chondroitin sulfate wound matrix [14,15], and investigated physical characteristics of the scaffold such as its mechanical strength and thermal stability, its resistance to enzymatic digestion, and the time needed to fibril formation [14,15,16]. The unique characteristics of the scaffold has addressed some of the disadvantages associated with commercially available injectable products, one being the lack of true flowability which may result in deficient filling of intricate wound beds, leading to insufficient integration and delayed wound healing.

Here, we evaluated the integration of the liquid scaffold with the host’s dermis and compared the results to those of a granular collagen-based product (GCBP) that is commercially available and clinically approved for the treatment of tunneled wounds.

The main aims of this study were to assess the time needed for scaffolds to incorporate into the surrounding tissue, and to identify the ensuing host reaction. Since the ECM is a component of the cutaneous tissue, both the liquid scaffold and GCBP would behave most naturally if they were applied in direct contact with the host dermis. In mice, a thin layer of muscular tissue, the panniculus carnosus, is strongly attached to the skin throughout the dorsum and any attempt to inject scaffolds subcutaneously would result in their application beneath the muscle layer and away from dermis. Therefore, we developed a microsurgical technique to dissect between the dermis and the panniculus carnosus to create sub-dermal pockets that we can inject the scaffolds into, ensuring they come in contact with the dermis.

Several studies and review papers have reported that advanced, injectable ECM matrices had limitations with early integration with the host tissue, delaying wound closure and increasing the risk of wound infection [12,13,24]. However, our results demonstrated that the liquid scaffold was associated with a rapid integration. it interacted with the host and created a microenvironment allowing cellular infiltration of mainly stromal, fibroblast-like cells, followed by immune cells. In addition, Liquid Scaffold promoted cellular proliferation as the number of these cells increased from week 1 to week 2. Despite evidence that suggests dermal implantation of biomaterials can induce a foreign body reaction (FBR) in the form of immune cells surrounding the periphery of the material; giant cells forming within the material; or a fibrous capsule formation [11,25,26,27,28,29], no adverse FBR was associated with the application of the liquid scaffold. Histological examinations after 1 and 2 weeks demonstrated well-organized interaction between the matrix and the surrounding tissue, with cells infiltrating within the matrix rather than surrounding it, where most of these cells were identified to be Vim+ stromal cells, rather than CD45+ immune cells that classically dominate the cellular invasion involved in FBR to implanted ECM scaffolds [11,25,26,27,28,29]. Additionally, there were no visible collections of fused CD45+ cells in the liquid scaffold, which is evident of the absence of a foreign body giant cell formation reaction. Lastly, no sign of a rejection towards liquid scaffold was observed, as the number of CD3+ T-cells present at all time points did not significantly differ from that seen in the control group (*p* > 0.05). These characteristics contrasted what was observed following the injection of the commercial GCBP. A dense, capsule-like cellular layer of mainly Vim+ cells surrounded the matrix (Figure S1), resulting in restricted cellular infiltration throughout the 2-week duration of the study. This delayed incorporation has been previously reported in ex vivo and in vivo studies investigating the biocompatibility of the product [12,30].

## 3. Conclusions

Although both scaffolds are mainly composed of collagen and a GAG component, each exhibited a distinctive microstructure and induced a different host response, resulting in varying integration patterns. Our results showed an early and efficient integration of our scaffold, accompanied by significantly higher cellular infiltration of Vim+ after 1 and 2 weeks, and CD45+ cells after 2 weeks.

Results obtained here give us new insights into the characteristics of in-vivo application of our wound matrix, and supplement existing knowledge about the host response to the application of the commercial GCBP. Further research is needed, however, to advance our current understanding of the reparative potentials of both scaffolds. Future works will investigate specific cell phenotypes involved in the host response, including fibroblasts and the pro- and anti-inflammatory subtypes of macrophages. In addition, the potential of each scaffold to promote new vessel formation will be assessed by monitoring in-vivo vascularization.

## 4. Materials and Methods

### 4.1. Preparation of Scaffolds

Cross-linked collagen-chondroitin sulfate wound matrix (Liquid scaffold) was prepared in sterile conditions as previously reported [14,15]. Briefly, bovine type I collagen (Advanced Biomatrix, Carlsbad, CA, USA) and chondroitin-6-sulfate (Sigma Aldrich, Oakville, ON, Canada) were combined (1:6 *w/w*) to a final concentration of 3 mg/mL collagen and neutralized with DMEM and 1N NaOH. Glutaraldehyde (0.02% *v/v*; Sigma Aldrich, Oakville, ON, Canada) was used to crosslink the collagen for 1 h on ice in the dark. After cross-linking, glycine was used to de-activate residual aldehydes. After washing, PVA (50:50/208,000 and 146,000 MW, 0.2% *w/v*; Sigma Aldrich, Oakville, ON, Canada), sodium borate decahydrate (0.05% *w/v*; Sigma Aldrich, Oakville, ON, Canada), and ascorbate (pH 7, 100 μM; Sigma Aldrich, Oakville, ON, Canada) were added to the cross-linked collagen to construct the final scaffold. Scaffold was kept at 4 °C in liquid form until it was ready to use on animals. Granular collagen-based product (GCBP) is commercially known as Integra^®^ Flowable Wound Matrix (Integra Lifesciences, Princeton, NJ, USA). GCBP was prepared according to the manufacturer’s instructions, by thoroughly mixing a syringe containing 3 cc of micronized collagen-GAG with 3 mL of sterile saline until the slurry matrix was formed, and then sub-dermally injected in animals.

### 4.2. Animal Handling and Anesthesia

All animals were treated humanely in accordance with approved protocols by the Animal Care Committee at the University of British Columbia (Protocol #A14-0309) and in compliance with the Canadian Guidelines on Animal Care. Female Balb/C mice (Charles River Laboratories, Bar Harbor, MD, USA) were housed and maintained in a clean facility, and surgical procedures were carried out in sterile conditions. Six 12-week-old mice, between 18 and 20 g, were anesthetized with 5% isoflurane in 100% oxygen at 2–4 L/min for induction, followed by maintenance with 2% isoflurane. Dorsal hair was removed using clippers and Nair^®^ depilatory cream, then the back was prepped with povidone-iodine and 70% alcohol. 5 mg/kg Meloxicam (Boehringer Ingelheim Animal Health, St. Joseph, MO, USA) was injected subcutaneously prior to surgery and for 3 days post-operatively.

### 4.3. Surgical Procedure and Scaffolds Application

**Figure 7:**
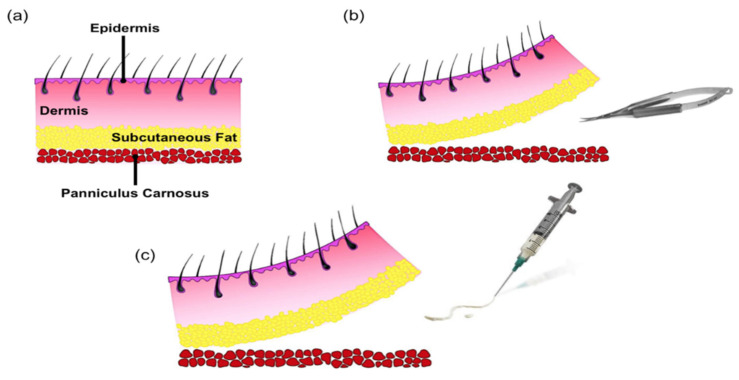
Diagram depicting the microsurgical technique used in the study. (**a**) Intact mouse skin. (**b**) Subcutaneous pockets were created by dissecting between the dermis and the panniculus carnosus muscle layer. (**c**) Each pocket received 200 μL of Liquid Scaffold, GCBP, or sterile saline (sham control).

To ensure the application of the scaffolds in direct contact with the mouse dorsal dermis, surgical microscope ZEISS OPMI 6-SD on Universal S3 stand (Zeiss, Toronto, ON, Canada), and a microsurgery set (S&T, Neuhausen, Switzerland) were utilized to dissect between the dermis and the underlying panniculus carnosus muscle layer. First, three 8 mm incisions were made on the back of each mouse, and dissecting scissors were used to separate the epidermis and dermis from the underlying, strongly attached panniculus carnosus to generate three separate sub-dermal pockets (Figure 7). Each pocket received 200 μL of liquid scaffold, GCBP, or sterile saline (sham control). Incisions were primarily closed with 6-0 non-absorbable nylon sutures (Stevens Company, Burnaby, BC, Canada). Finally, Tegaderm™ Film dressing (3M Healthcare, Toronto, ON, Canada) was used directly on the back to protect the wounds, then secured with a layer of Co-Flex self-adherent wrapping (Andover Healthcare, Salisbury, MD, USA). Samples were harvested after 1 and 2 weeks (*n* = 3 per week, total *n* = 6).

### 4.4. Histological Evaluation

To evaluate the interaction between scaffolds and the host, tissues were harvested and immediately fixed in 10% neutral buffered formalin. Paraffin-embedded sections were cut at 5 µm and stained following the standard Masson’s Trichrome (MT) staining protocols. MT stains collagen fibers blue, cytoplasm and nuclei light pink and dark brown, and muscle fibers red. Stained slides were scanned with the ScanScope CS system (Aperio, Vista, CA, USA).

### 4.5. Immunofluorescence Staining and Cellularity Quantification

Immunofluorescence (IF) staining was performed to further assess and quantify cellularity. Staining and quantification were conducted for cytoplasmic Vimentin (a marker of cells of mesenchymal origin, and an established general marker of dermal fibroblasts), cell-membrane CD45 (a marker of differentiated hematopoietic cells such as macrophages, B- and T-lymphocytes), and cell-membrane CD3 (a general marker of T-lymphocytes). Samples were harvested and fixed in 10% neutral buffered formalin. Paraffin-embedded sections were cut at 5µm and stained following standard IF staining protocols. For intracellular Vimentin staining, heat-induced antigen retrieval was performed with sodium citrate buffer solution, followed by membrane penetration with 0.05% saponin in water for 5 min, then washed three times for 5 min with phosphate buffer solution containing 0.1% tween 10 (PBS-T [pH 7]). Non-specific antibody binding was blocked with 5% bovine serum albumin (BSA) in PBS-T for 1 h at room temperature. After washing, Rabbit Anti-Vimentin antibody (ab92547; Abcam Inc., Toronto, ON, Canada), 1:100 in 2% BSA in PBS-T, was added and slides were incubated overnight at 4 °C. After washing out primary antibody, secondary Rhodamine Red™-X (RRX) AffiniPure goat anti-rabbit IgG (H + L) antibody (#111-295-003; Jackson ImmunoResearch Laboratories Inc., Burlington, ON, Canada), 1:750 in 2% BSA and 2% normal goat serum (NGS) in PBS-T, was used for 1 h. Nuclei were stained with Fluoroshield Mounting Medium with DAPI (ab104139; Abcam Inc., Toronto, ON, Canada). For cell-membrane CD45 and CD3, similar antigen retrieval step was performed, followed by washing with PBS (pH 7) then blocking with 5% BSA in PBS for 1 h. Slides were then separately incubated with Rat Anti-CD45 antibody (30-F11; Invitrogen, Thermo Fisher Scientific, Waltham, MA, USA) and Rabbit Anti-CD3 antibody (ab16669; Abcam Inc., Toronto, ON, Canada), 1:100 in 2% BSA in PBS overnight at 4 °C. Secondary antibody incubation with Rhodamine Red™-X (RRX) AffiniPure goat anti-rat IgG (H + L) antibody (#112-295-003; Jackson ImmunoResearch Laboratories Inc., Burlington, ON, Canada) and Rhodamine Red™-X (RRX) AffiniPure goat anti-rabbit IgG (H + L) antibody (#111-295-003; Jackson ImmunoResearch Laboratories Inc., Burlington, ON, Canada), 1:750 in 2% BSA and 2% NGS in PBS for 1 h, were used for CD45 and CD3, respectively. Again, nuclei were stained with DAPI. Tiled images were taken using a ×20dry objective of a Zeiss AxioObserver Z1 confocal microscope fitted with a CSU-X1 spinning disc (Yokogawa Inc., Calgary, AB, Canada) and AxioVision 4.8 (Zeiss, Toronto, ON, Canada), and analyzed using Zen 2 software (Zeiss, Toronto, ON, Canada). Quantification was performed by counting the number of cells in each of the ten randomly selected, high power field (HPF) areas across each independent sample section, using Z-stacked images with a ×40 wet objective of the same confocal microscope. Images were analyzed using the Zen 2 software. For final quantification, the 10 HPF areas per independent sample were averaged over triplicate.

### 4.6. Statistical Analysis

All experiments were performed in triplicate (*n* = 3). Data shown are means ± standard error of mean (SEM). Differences in means among groups were tested using one-factor ANOVA with Tukey post-hoc analysis (GraphPad Prism software version 7). *p*-values < 0.05 were considered statistically significant. This significance is indicated with an asterisk.

## Data Availability

The data presented in this research are available in the article or from the corresponding author upon reasonable request.

## Supplementary Materials

The following are available online at https://www.mdpi.com/article/10.3390/gels8010049/s1, Figure S1: Immunofluorescence staining of the cellular capsule surrounding GCBP during the first two weeks following application.

## Abbreviations

ANOVA	Analysis of Variance
BSA	Bovine serum albumin
ECM	Extracellular matrix
FBR	Foreign body reaction
GAG	Glycosaminoglycan
GCBP	Granular collagen based product
H&E	Hematoxylin and Eosin
HPF	High power field
IF	Immunofluorescence
IgG	Immunoglobulin G
MT	Masson’s Trichrome
NGS	Normal goat serum
PBS	Phosphate buffered saline with Tween 20
PVA	Polyvinyl alcohol
SEM	Standard error of the mean
VIM	Vimentin
