# Supplementary material for: Evaluating the Biocompatibility of an Injectable Wound Matrix in a Murine Model
Source: Gels. 2022 Jan 9;8(1):49. doi: 10.3390/gels8010049 (PMC8774422; doi:10.3390/gels8010049)
Supplement: Supplementary file 1 [file gels-08-00049-s001.zip › gels-1500234-supplementary.pdf]

Supplementary Material:

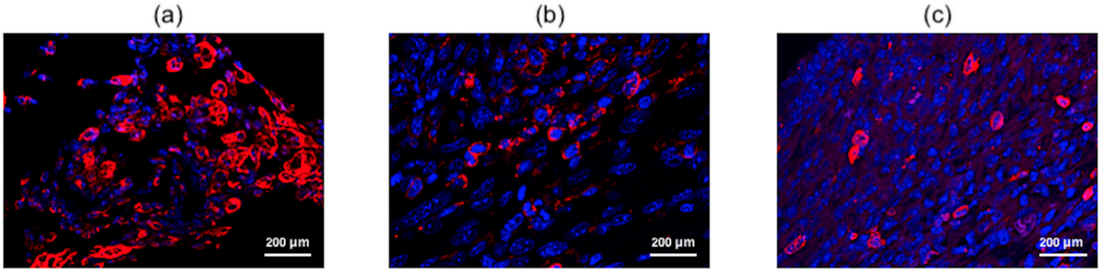

Supplementary Figure S1. Immunofluorescence staining of the cellular capsule surrounding GCBP during the first two weeks following application. (**a**) Vim+, (**b**) CD45+, and (**c**) CD3+ cells. Scale bars = 200 µm.
